# Older Clients’ Pathway through the Adaptation System for Independent Living in the UK

**DOI:** 10.3390/ijerph17103640

**Published:** 2020-05-21

**Authors:** Wusi Zhou, Adekunle Sabitu Oyegoke, Ming Sun, Hailong Zhu

**Affiliations:** 1 School of Public Administration, Hangzhou Normal University, Hangzhou 311121, China; zhhl59@126.com; 2 School of Built Environment & Engineering, Leeds Beckett University, Leeds LS1 3HE, UK; A.Oyegoke@leedsbeckett.ac.uk; 3 School of Architecture, Design and the Built Environment, Nottingham Trent University, Nottingham NG1 4FQ, UK; ming.sun@ntu.ac.uk

**Keywords:** housing adaptation, older people, aging in place, environmental gerontology

## Abstract

Housing adaptation is recognized as an effective intervention for successful independent living and has been given a greater political priority. However, the current adaptation implementation is fragmented and sometimes confusing. This study is aimed at examining blockages in the adaptation system in the United Kingdom (UK) and identifying practical ways to tackle them. It adopted a mixed-method sequential explanatory research strategy. A questionnaire survey was first conducted in all local authorities in England, Scotland and Wales. This was followed by individual interviews and a focus group with professionals and older clients. The study found that multiple organizations are involved during the delivery of housing adaptations; poor cooperation between them is a major barrier to a seamless service. The adaptation process involves five key stages; there are many inconsistencies and inequities in the process across local authorities. Significant delays are found at all stages, the average length of time taken to complete an adaptation is unacceptably long. There are also many inconsistencies and inequities across different local authorities. This study identified some common deficiencies, which cause inefficiencies and ineffectiveness in housing adaptation practices and makes some recommendations on specific actions that need to be taken at both national and local levels to address them.

## 1. Introduction

One distinguishing characteristic associated with old age is the deterioration in functional abilities caused by diabetes, osteoporosis, sensory impairment and other health problems [1,2,3]. Long-term disability often results in older people facing environmental barriers at home, such as those that stop them from reaching the toilet, having a bath or going upstairs [4,5,6]. More worryingly, older people are more likely to live in homes that are in poor condition [7,8,9]. Unsuitable housing conditions can bring about preventable falls and an increased risk of life-limiting illness experienced by older people [10,11]. According to the World Health Organization (WHO) [12], falls at home are the prominent cause of injury or ill health among older people and every year approximately 35% of people aged 65 or over experience falls. Therefore, housing quality and suitability are fundamental determinants of health and well-being [13,14]. Bull and Watts described factors related to the specifics of home injuries [15], whereas the International Classification of Functioning, Disability and Health (ICF) defined the broader principle of environmental factors mediating the extent to which functional decl ine results in disability [15]. For example, a person with osteoarthritis may or may not experience a disability dependent on the extent to which their living environment supports or hinders their physical activity and social participation [16]. In this regard, housing adaptation, defined as a modification of physical features in the indoor and immediate outdoor environment to remove physical barriers, is a foundation for successful independent living.

Until thirty years ago, the living environment of senior citizens was largely overlooked [17]. This problem was partially corrected by a growing number of environmental gerontology studies [18,19,20] which focused on the role of the physical environment in the lives of older people. Environmental gerontology investigates the person–environment fit processes in aging from an interdisciplinary perspective [21,22], with the central aim of optimizing the relationship between an aging person and their physical-social environment [23] and of seeing a society that better meets the needs of an aging population [24,25]. The ecological theory of aging, developed by Nahemow and Lawton [26], is recognized as one of the most referenced and prominent theories in the field of environmental gerontology [27,28,29]. It involves two valuable concepts of personal competence and environmental press [30]. Specifically, the theory suggests that a person with low functional competence is more vulnerable to environmental influences than a person with high competences [30,31]. There is an optimal person–environment fit when personal competence is compatible with environmental demands [32,33]. In other words, adapting the environment can help less competent people improve their functional performance to match environmental demands [19,34].

General ecological theories have offered a better understanding of the relationship between people and the environment. In practice, adapting the house to help older people remain independent could be beneficial to individuals, families and governments. A number of studies [35,36,37] have reported that housing adaptations have improved health, independence and quality of life in later life. Beyond health and independence, several other studies [38,39,40] have further found that environmental adaptations have reduced older people’s need for support from family caregivers and have lessened the strain on family relationships. Because of their key role in preventing falls, avoiding hospital admission and reducing domiciliary care, housing adaptations have brought significant savings to the National Health Service (NHS) services, home care costs and social services budgets [41,42,43]. In the UK, local governments have the statutory duty to fund housing adaptations for disabled people. There are differences in the types of financial assistance for adaptations in different housing tenures. Homeowners and private sector tenants apply for specific adaptation grants, e.g., disabled facilities grants (DFGs) in England and Wales and mandatory grants in Scotland, while social housing tenants use alternative funding from their landlords, e.g., housing revenue accounts and housing association funding. According to the Housing Grants, Construction and Regeneration Act 1996, DFGs are mandatory when adaptation work is necessary and appropriate to meet the needs of disabled people and when it is also reasonable and practicable considering the property’s age and condition. During the assessment process, the housing department should consult the social service department about the necessity and appropriateness of the adaptation. As a result, the adaptation process involves at least two local authority departments, with the social service carrying out assessments and the housing providing grants.

Despite the importance of a seamless service for providing timely assistance and overcoming environmental barriers, very few studies [44,45] have focused on reviewing the process of housing adaptations and improving their effectiveness. There is an urgent need for investigating and assessing the current practice of delivering housing adaptations in the UK. This study is aimed at filling the knowledge gap by conducting a sequential mix-method research on this topic. An earlier paper in this journal reported findings of the timeliness across the key stages of the housing adaptation service and the main causes of delays in current practice [46]. This paper explores, in more depth, the challenges for the coordination of complex pathways to adaptation services. Its main objectives are: (i) to identify how local authorities organize and manage their adaptation services; (ii) to examine common issues with the pathway in which adaptations are delivered and explore why they occur; (iii) to make recommendations for improvement to the adaptation system at both national and local levels.

## 2. Methods

### 2.1. Research Strategy

**Figure 1:**
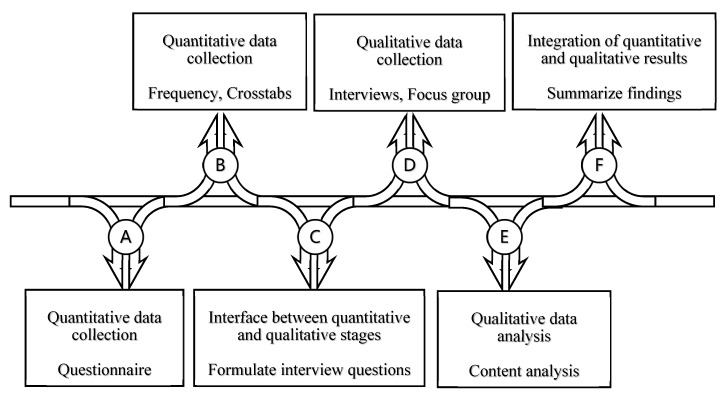
The flowchart of conducting this mixed-method research.

This study employed a mixed-method approach to investigate the blockages that prevent the flow of the adaptation process in different regions of the UK. It chose to focus on homeowners and private tenants instead of public sector tenants, as these two groups account for the majority of households in the UK and most of them have little knowledge about where to seek help for adaptations. A sequential explanatory strategy was adopted, in which the quantitative and qualitative processes come in two consecutive phases with six steps (Figure 1). The first phase used a survey which was designed to understand how local authorities organize and manage their adaptation services. The second phase included interviews and a stakeholder one focus group, involving social workers, occupational therapists (OTs), housing officers, staff from other agencies and older service users. The aim of the second phase was to explore different perspectives on the statistical results in more depth. Interviewees were selected from survey respondents and interview questions were formulated based on an analysis of survey results. Findings from the two phases were finally integrated to form a comprehensive insight on the current practice of housing adaptation in the UK.

### 2.2. Data Collection

Following a systematic review of policy documents, research reports and journal articles, the questionnaire survey was carried out in 2015 with all local authorities in England, Scotland and Wales. County councils in England were excluded, as they are not involved in organization of housing adaptations and they only provide OT assessments. Local authorities in Northern Ireland were also excluded, as the region has unique health and social services under a unified structure and delivers adaptation services that are different to the other nations in the UK. Before the main survey, a pilot was carried out with 20 local councils to ensure the clarity and appropriateness of all the questions. The finalized questionnaire, along with a cover letter and a prepaid envelop, was sent to 378 local authorities in England, Scotland and Wales. At the same time, an online version of the survey was activated to provide an alternative response method. Reminder phone calls and emails were made after four weeks to local councils who had not responded. In the end, a total of 112 responses were received, including 61 by post, 28 online and another 23 by emails, with a response rate of 29.6%.

After the survey, interviews and a focus group were carried out in order to understand local arrangements for delivering adaptations and any concerns with these arrangements from the perspective of service providers and service users. Five professionals were selected from the survey respondents. They worked in different organizations or departments and were responsible for different stages of the adaptation process. Interviews were conducted in interviewees’ offices and lasted for 60 to 150 min. Interview questions were open-ended and focused on the delivery system from service planning to service performance in local authorities. Two clients were identified during these interviews with professionals; they were aged 65 or over and received housing adaptations in the last two years. They were interviewed in their homes for around 60 min each, with the aim of exploring their experiences and concerns on key aspects of the journey of housing adaptation. These interviews provided a direct insight into the client perspective of characteristics and deficiencies in current adaptation practices. For a deeper understanding of blockages within the adaptation system, a focus group meeting including an OT, two housing officers, a Care and Repair (C&R) manager, a technical officer and a coordinator was organized in one local authority. This local authority has the social work, the housing department and C&R working in partnership during the delivery of housing adaptations. All interviews and focus group discussion were recorded and transcribed verbatim afterwards.

### 2.3. Data Analysis

An exploratory quantitative data analysis approach was employed to look at individual variables and their components in the initial analysis stage. Survey responses were coded and tested by using SPSS to identify how housing adaptations were organized. Descriptive statistics were used to examine similarities and differences between local adaptation practices. Bivariate and multivariate analysis was carried out to examine relationships between variables and to determine whether any association is significant. NVivo was used to analyze data from interviews and the focus group. Thematic analysis was adopted to identify, analyze and report patterns within these qualitative data.

### 2.4. Ethics

This study was conducted in line with the ethical principles of not causing harm, obtaining informed consent, respecting privacy and avoiding deception. Potential ethical issues were evaluated and discussed; all participants were informed of all aspects of the research and gave their consent.

## 3. Results

### 3.1. Multiple Partners

The survey sought to establish the prevailing organizational structure adopted by local authorities to deliver housing adaptation. Respondents were asked to indicate the key partners in their areas and whether they adopted any written guidance for the process. They were also asked to rate the effectiveness of their current practice. Table 1 shows the survey results.

The current legal system for the delivery of housing adaptations in the UK is complex, with the social services department responsible for assessment and the housing department responsible for grant approval. Table 1 shows that the two departments were found as the key partners for the provision of adaptations in 74.1% and 83% of local authorities respectively. When the Local Government and Housing Act 1989 gave local authorities the power to provide financial assistance for agency services, home improvement agencies (HIAs) and C&R became significant in supporting older people through the adaptation process. The survey showed that 75 out of 112 local councils have worked with external associate agencies such as HIA and C&R in carrying out housing adaptations. Many agencies offered information and advice on funding and other services, such as producing specifications, securing quotes and supervising installations. An interviewed manager of C&R said:

“We are independent and flexible to provide services through the whole process from referral to completion. For example, the grant application needs the title deed, the approval of building insurance, the approval of all the client’s incomes, and the letter from the mortgage provider if the client still has mortgage; older people have no idea how to prepare them. We can go to the client’s house with a big pile of paperwork and help them to go through what it is looking for.”

These agencies provided quality care support for the elderly and placed them at the center of adaptation services, as highlighted by a C&R officer:

“We can spend time with clients and help them through the whole process. This is really care, not just repair. Care is a very important part, because getting adaptations done can be very stressful for older people. This is why most C&R are quite successful.”

It is found that older people were largely satisfied with the agency service, which helped achieve their high expectations of what is to be delivered. One interviewed older client commented:

“At the beginning I had some worries about the shower and the toilet seat, which come out around thousands of pounds and need certain building work. Care and Repair got everything done for me. Brilliant!”

Despite this, in many local authorities, HIA and C&R were not treated as strategic partners when planning and developing adaptation services. Such an arrangement limits the agency’s potential to improve the efficiency of adaptation systems and to achieve a greater flexibility of using the available resources. An HIA officer said:

“As the exact procedures and resources for adaptations are decided by the council, we are not able to use innovative ideas to improve the process or save the budget. We can just do what we have been told.”

The integrated authority, as a solution to fragmented responsibilities, has not received much attention. Only 15 local councils have established an integration authority as a single body responsible for adaptation services. Some local authorities also worked with other organizations such as the NHS and social enterprises. Overall, the process of adaptation was administered by different groups in different local authorities. Their cooperation was the key for a seamless service. To develop effective joint work, 79.1% of local authorities have published guidance on the responsibilities and duties of partners. Most local authorities were happy with their current delivery systems; 91.7% of respondents considered their current partnership as fairly effective or very effective. However, there were still conflicts and disconnections between the partners, especially when the adaptation process was organized under a two-tier system. One grant officer reported:

“The enquiry and assessment processes are managed by the county’s occupational therapy staff, the local authority has no involvement in this other than signposting.”

To end the fragmented responsibilities across multiple local authorities, a housing officer sought a location-based service:

“Having one dedicated team based in one location would be beneficial to reduce waiting times and streamline the process.”

Another housing officer further suggested a standard (Information Technology) IT system for better information sharing:

“Better alignment of IT system enables all partners to transfer information and to monitor cases effectively.”

However, most local authorities did not have an information system that can be accessed by all partner organizations, as criticized by a C&R officer:

“No, we don’t have a shared system and we suppose we should have more information. We don’t have any access to the council system, we have our own system. So it just wasn’t practical.”

The lack of a shared system made it difficult for service providers to track the progress of cases and to provide updates to clients. The C&R officer continued:

“Because there isn’t a single shared system, we don’t know how long the process takes and which stage the case is at. We can only advise to the council if we have any problems on our own.”

### 3.2. Fragmented Process

**Figure 2:**
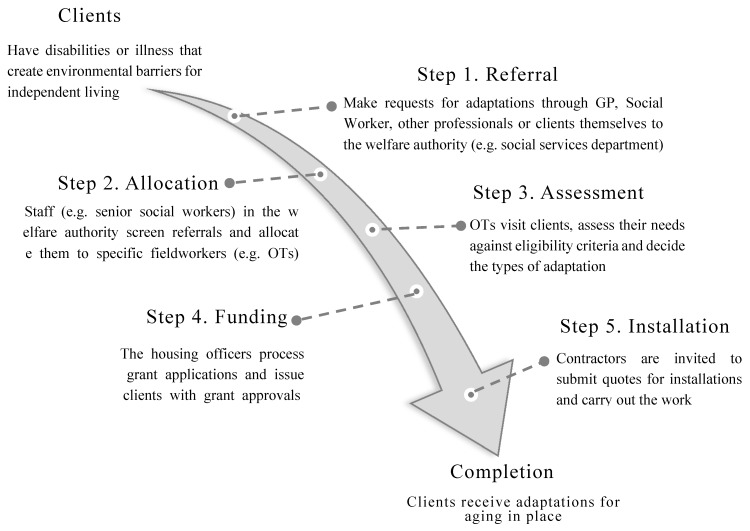
The pathway through the adaptation system.

In addition to the involvement of multiple partners, the housing adaptation process also involves multiple stages. Figure 2 shows the typical pathway of the process and its major steps.

Because of imprecise legislation and fragmented responsibilities, a client has to navigate the service pathway through a series of developmental stages when applying for adaptation support, including referral, case allocation, need assessment, funding authorization and installation (Figure 2). Where an adaptation is required, the initial enquiry is usually made to the social services department in a local authority. Often, referrals come from a range of sources, such as health professionals and applicants themselves, as explained by a housing officer and an older client during the interviews:

“People are often unclear who they need to approach when they require an adaptation, so the route to referral can vary–they approach their housing provider, they visit their General Practice (GP) doctor, they are referred by a health professional whilst in hospital, they are referred by hospital occupational therapist, they contact their local authority, they contact their social worker, or they ask a relative or carer.”

“If I have a problem, I will first contact my doctor, they can get referrals through consultants. My doctor will speak to consultants in hospitals and they will then put me in touch with specialists in the council.”

Thus, it is important that all access points have a standard inquiry form or a shared system to collect basic information for need assessment, as commented by an interviewed social services officer:

“Any enquiries made directly to our council are usually referred to social services on the same day when a standard form provided by social services.”

However, the survey found that 38.9% of responding local councils did not adopt any of these standard approaches. As a result, clients’ requests for adaptations may have taken longer to reach OTs or could not be processed equally and consistently.

On receipt of referrals, an initial screening process normally takes place to prioritize cases and allocate them to specific fieldworkers, mainly OTs, for assessments. The survey showed that 79 out of 112 local authorities had used an initial screening mechanism, as said by a social worker:

“Our staff and seniors on a regular basis screen the initial enquiries and then decide what priority they fit into.”

This screening system enables local authorities to track urgent needs and to focus on more complicated requests. An OT further added:

“A priority scoring system gives us the opportunity to deal with the most complicated situations and to visit high priority cases more quickly.”

However, most local authorities did not set target timescales for assessments of different priority categories, resulting in non-urgent applicants waiting for even longer. A housing officer reported:

“There are lengthy waiting lists to be assessed by occupational therapists especially if the adaptation isn’t seen as critical by the local authority.”

Furthermore, due to limited financial resources, most local authorities used various eligible criteria to decide high priority adaptations, as highlighted by a grant officer and a C&R officer:

“The available budget is only sufficient to resource critical and substantial cases living in private sector housing.”

“Based on the client’s needs each case is prioritized and given a number of points 6, 7 up to 13. Most clients are 11 or 12 points. So for those with 8, 9 or 10 points, there is really no chance for them.”

Evidence shows that adaptation services often focus on urgent cases rather than anticipating needs; the current approach is still reactive rather than proactive. However, an earlier intervention can support low priority clients to maintain independence for longer and bring cost savings to NHS services and social services budgets. A social worker claimed:

“We provide adaptations to people in priority 3 or 4 (low level category priority). The reason we do that is prevention. We provide these people with adaptations, so that they can manage to get downstairs, hopefully, without falls. For a long time, this will save lots of money and improve quality of life for these people, meaning they are more likely to keep active, not get worse, and not have accidents.”

After assessment, the case is passed to the grant officer in the housing department for funding approval. As mandatory adaptation grants like DFGs are contributory, there has to be a means test for all applications, except for those from children or young people. To pass this means test, the applicant needs to provide a range of documents, such as bank statements, pension books and proof of other benefits. Collating and preparing these documents can put great strain on older people and can be time-consuming, as pointed out by a C&R officer:

“When we tell older clients that we need these documents, in most cases, they are scared and don’t know what they are going to get. So the process would be a lot slower.”

When a grant application is made by a tenant, there is a need to obtain consent from the landlord. No work can be carried out on a property until the local authority receives the written permission from the landlord. A housing officer reported:

“Delays mainly result from obtaining landlord permission for private rented tenants and social tenants.”

Even worse, some landlords were unwilling to give their permission for home adaptations and as a result tenants had to move out, as reported by a C&R and a housing officer:

“We have had some occasions, where the landlord would not allow the adaptation to go ahead, so the tenant had to find another place. That’s a shame.”

“Sometimes the landlord would rather ask an elderly person to move than adapt a family home for him or her.”

To prevent this, a good practice is to have a discussion with the landlord as early as possible and provide an offer to restore the property to its original condition or sell the property to the council when the tenant moves elsewhere or dies. However, this practice has not been shared widely, as noted by a C&R manager:

“There should be the option and some money to put the home back when a tenant no longer requires the adaptation, but I have never heard of it. This doesn’t happen in our council, and most of councils won’t put money in this area.”

Once a grant has been approved, installation work can go ahead. Firstly, the client’s needs must be translated into a specification as a schedule of works. It is usually the result of cooperation between several professionals, such as the OT, an architect and a grant officer. A housing officer and a C&R manager said:

“Where we have a direct involvement, we arrange to meet the OT and the contractor on site to discuss the specification of the installation.”

“The OT, the technical officer and the grant officer will visit the site together to decide the specifications of the adaptation.”

When plans and specifications for the adaptation work are finalized, the client starts to seek quotations but frequently faces difficulties in finding appropriately skilled contractors. To assist clients, the survey found that 78 local councils maintained a list of approved contractors. This was confirmed by a C&R officer:

“We have a list of trusted contractors and have built good relationships with them over ten years.”

This approved list helped clients get the requisite number of quotes in a timely and efficient manner, as described by an older client:

“C&R suggested the names of a few contractors on their list and got quotes from them. They provided me with the three least expensive ones because of my funding, then I picked.”

More importantly, these accredited contractors usually completed the work to a high standard, as reported by an agency officer:

“Every adaptation met our expectations and most of clients were happy with the contractor’s work.”

However, some local authorities have not yet compiled such a list of contractors even though they realized the need for it, as put by a grant officer:

“To minimize waiting times for adaptation, we need more technical staff to assist clients with their enquiries and more suitable building contractors.”

### 3.3. Waiting Timelines

**Figure 3:**
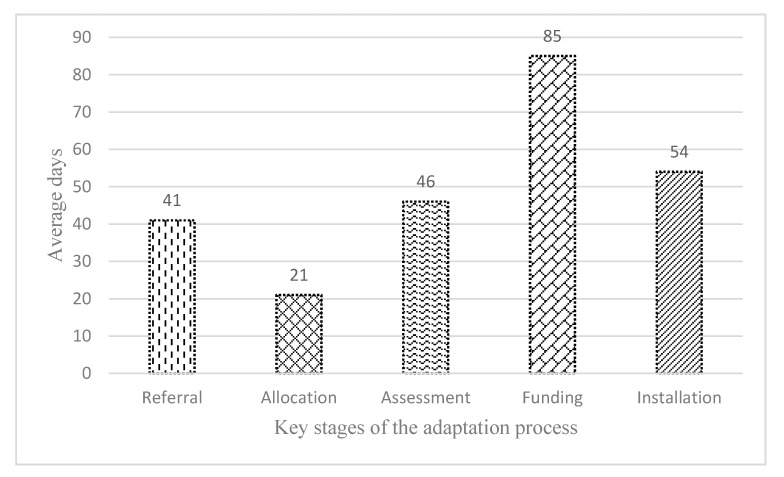
Average waiting time for each stage in the adaptation process.

Getting an adaptation done successfully requires a range of tasks to be completed in sequence in five key stages, including referral, allocation, assessment, funding and installation. Figure 3 compares the average length of time for each key stage in the whole adaptation process.

Overall, the average waiting time for each stage was often unacceptably long. The longest wait of 84 days occurred at the stage of funding authorization, which accounted for 34% of the total 247 days. This was mainly caused by unexpected demand in conjunction with limited resources:

“Demand for adaptations exceeds financial resources, which means a waiting list for DFGs.”

(a housing officer).

Besides, after means testing, some clients were required to make a financial contribution towards the cost and had to spend time securing additional funding when they could not afford the contribution, as highlighted by a housing officer:

“Delays can often occur when clients are required to find the necessary resources towards a funding contribution or share of the costs.”

Otherwise, there is no chance for them to receive adaptations, as explained by a C&R officer:

“If that is the case that the clients only get 80% of the cost, we have to try to raise the money through other ways. Because the clients have a low income, they probably would not have access.”

The second longest average waiting time of 54 days was found at the installation stage, which was mainly caused by legal procedures, such as planning permission and the timing of installation. For some major adaptations (e.g., an extension, a structural alteration), clients had to apply for planning permission or building control approval, which was time-consuming and expensive, as remarked by a C&R manager:

“These legal requirements need to follow; I can understand that. It is just annoying that they add a number of weeks to the process.”

According to the Housing Grant, Construction and Regeneration (HGCR) Act, clients are eligible to carry out adaptation work within twelve months from the date of grant approval, as explained by a housing officer:

“When somebody has been offered the grant, by law, they have up to one year to spend that and don’t have to start the works straight away.”

It was found that clients frequently put the installation process on hold and delay the building work after grant approval is received. The survey found two opposite ways to deal with the unspent grants—to withdraw or carry forward. Most local authorities set a deadline within which the grant should be spent, otherwise it is be withdrawn unless there are proven extenuating circumstances:

“DFGs are valid for 12 months following approval. Cases that exceed this period are reviewed on a case by case basis and will only be extended if there are reasonable causes.”

(a housing officer).

On the other hand, some local authorities allowed the grant to be carried forward to the following financial year if it was not spent within the current financial year. It all depended on the circumstances of the applicant:

“Applicants have an initial 12 months to use their grant award. If it is not spent within that period, the council will discuss options with the client to extend the time period and to assist them in taking the project forward.”

(a housing officer).

Clients often experienced substantial delays at the assessment stage and the referral stage, which took an average of 46 days and 41 days respectively. The leading cause of slow assessment was the shortage of OT resources, as explained by a policy officer:

“There are huge variations in the number of occupational therapists per population in each area. The waiting lists for assessment vary considerably.”

To free up OTs, some local authorities established a web-based mechanism for self-assessment, as reported by a housing officer:

“We use self-assessment models for low level adaptations to allow greater capacity for OT staff to deal with cases in a reduced time frame.”

Some local authorities deployed OT assistants or other assessors, as explained by a housing officer and a social worker:

“The use of OT assistants for less complex case is effective to alleviate delays in getting assessments.”

“We train staff from housing, social work and NHS who can carry out assessments for simple requests, while professional assessments are undertaken by OTs for people who have more complex needs. This is effective in controlling the waiting lists.”

One major problem with the referral system was the lack of a standard inquiry form or a shared IT system to collect the basic information needed for assessment. Due to incomplete information, initial requests for adaptations may have taken longer to reach OTs or may even have been put aside, as put by a social worker:

“We often get poor information. A worst case scenario is, a GP sees an older person and would say, Miss XX is really struggling and needs an OT assessment. But we don’t know how urgent the case is and we don’t know in which way the person is struggling with, so she is put in the waiting list.”

### 3.4. Delivery Outcomes

**Figure 4:**
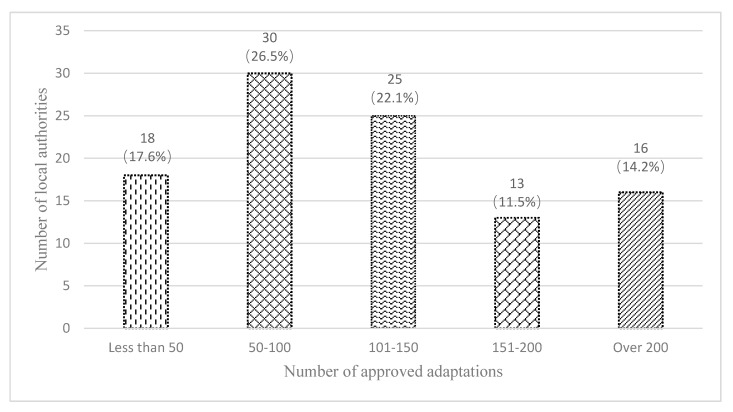
Local authorities completed different levels of adaptations.

Figure 4 divides local authorities into five groups according to the number of housing adaptations in an increment of 50, which they undertook annually.

The survey found that the average number of adaptations completed by one local authority in 2014/15 was 154, with a wide range of 20 to 1545. As shown in Figure 4, only 14.2% of 102 local authorities completed more than 200 adaptations, while 71.6% carried out less than 150 adaptations, which was a relatively small number compared with the potential needs of an aging population. This was confirmed during interviews with a housing officer and an agency manager:

“More and more older people demand adaptations, which always outstrips supply in our council.”

“Compared with the number of completed adaptations, the demand is far higher, because the population is aging.”

**Figure 5:**
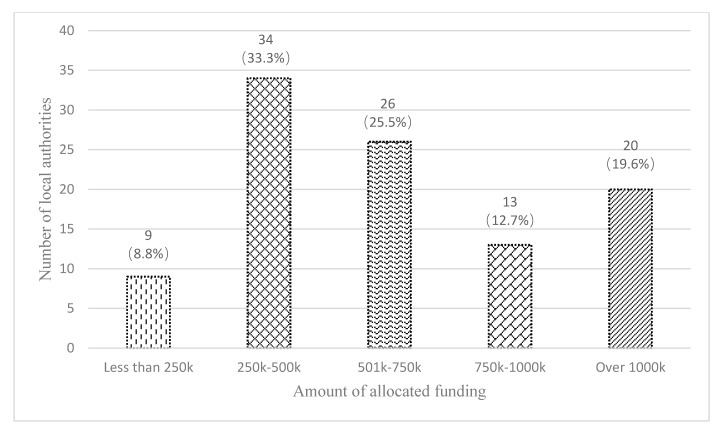
The number of local authorities at different levels of spending.

Also, there were significant variations in the amount of allocated funds per local authority, from £125k to £4500k, with an average of £777k. Figure 5 shows that 69 out of 102 local authorities spent less than £750k, which was not so different compared to previous years, as confirmed by a grant officer:

“We had the same money in total since 2007, the funding has not gone up. In fact, it has gone down. Technically, it has gone down, because the living expense has gone up.”

There is evidence that available funding was not able to meet the increased demand for adaptations, as complained by a housing officer:

“The number of adaptations required are increasing year on year. The demand exceeds financial resources.”

In addition to financial constraints, the adaptation management system also has an impact on the number of adaptations carried out by a local authority. Analysis showed a positive correlation between the use of a self-referral service and delivery outcomes (Table 2). 55.6% of local authorities, who received over 75% self-referrals completed more than 150 adaptations. In contrast, of those who did not use any self-referrals, 65.2% delivered a small number of fewer than 100. The self-referral service allows applicants to rapidly start the adaptation process and helps local authorities reduce associated administration costs, as confirmed by a social worker:

“Most people waiting for adaptations are first asked to self-referral through an online system. This saves our staff lots of time and helps people to enter the service quickly.”

Similarly, an initial screening system can enable local authorities to track urgent needs and to provide effective responses to all enquiries. Table 3 showed a positive association between the use of initial screening mechanisms and delivery outcomes. There were 38.3% of local authorities that screen initial requests on a regular basis and delivered more than 150 adaptations, compared with no local councils who did not have the screening system and achieved a similar level of performance.

## 4. Discussions

Currently, there are multiple departments or organizations working together to carry out housing adaptations [47]. Poor cooperation between the partnering organizations is a major barrier to a timely and effective adaptation service delivery. There needs to be formal arrangements in place to bring together housing, social services and any other services that play a part in the adaptation process. Otherwise, blockages will occur; as Heywood pointed out, “if one department is not working well, the work of other departments is undermined and yet there may be no way to raise and resolve the issue” [48]. An effective liaison can be secured by regular meetings, joint trainings and other agreed procedures, so that partners can develop functional communications and information sharing on service entitlement and delivery processes. Such cooperation is the key to speeding up the process and delivering the best value. Besides this, local authorities should recognize the role of agencies such as HIAs, in organizing and managing adaptation work, and coordinate with them to save both time and costs during the installation phase.

The current management procedure process, which takes the applicant through a series of steps including referral, allocation, assessment, funding and installation, sometimes appears to be opaque and hostile with lengthy delays [49]. There were a range of major issues that affected each key stage of the adaptation process. At the referral and allocation stages, there were different routes to making referrals for adaptation services, such as through GPs and other agencies or through self-referral. Due to the lack of a standard procedure across all access points, some applications were stopped before they had even started. Introducing a standardized referral form or a shared IT system would be beneficial. It would help collect necessary information without the need for redirecting clients or putting requests on hold. Many local authorities adopted a screening mechanism to prioritize initial requests into different categories. This mechanism helps local authorities provide faster OT visits and assessments for urgent needs, but as a result non-urgent applicants generally have to wait much longer. To address this, it is important for local authorities to take a proactive approach and to set a target waiting times for the assessment of different priority categories. At the assessment and funding stages, the requirement for OT assessments varied, as some local authorities allocated all referrals to OTs while others only involved them for complicated cases. Given that OT resources are in short supply in many local authorities, the use of ancillary assessments, such as self-assessments and OT assistant assessments, can offer a practical solution to long waiting lists for OT assessments. Delays often occurred when clients had to make a contribution towards the cost of the adaptation work. There should be specific arrangements to assist clients with their monetary contribution, subject to means testing. At the installation stage, maintaining a list of approved contractors within the local authority enables clients to make a speedy choice of a preferred contractor. The additional administrative procedures for some major adaptations, such as planning permission and building approval, were time-consuming and expensive. Central government should review the legal framework governing the provision of grants and allow local authorities more flexibility in the ways they carry out housing adaptations. Current policy allows clients to carry out the building work within twelve months after grants are approved, although some clients often delay the installation process beyond the deadline for a variety of reasons. There needs to be a clear explanation of when the installation work should be started and of the circumstances under which the timescale can be extended.

When a blockage exists at one stage of the housing adaptation process, the whole provision breaks down and the client cannot receive their requested adaptation in a timely manner. Delays are often found in each stage of the adaptation process. However, there are huge variations in waiting times for different stages, with the longest waiting time being observed at the funding and the installation stages. There are many causes for delays, including limited resources, unavoidable legal procedures, the client’s control over the installation work and the lack of a shared IT system. These delays have damaged the overall effectiveness of housing adaptation service provision. The number of adaptations is relatively small in most local authorities compared with the potential demands and so is the level of existing funding. Extra sources need to be provided at both national and local levels, so that adaptation services can reach more people in need across the country.

Although this study identified bottlenecks within each key stage of the adaptation process, it had some limitations. Because different organizations and staff were responsible for different stages, it was difficult to get all these partners to respond to the survey. This again revealed the deficiency that some local authorities did not have shared systems to facilitate effective joint working for adaptation services. In addition, time did not permit detailed work to be carried out on the provision of adaptations for service users with special needs.

## 5. Conclusions

Existing issues, such as fragmented responsibilities, bureaucratic procedures and lengthy waiting times, cause inefficiencies and ineffectiveness in housing adaptation services. These deficiencies show that fundamental changes are needed to reshape the organizational and financial systems both locally and nationally. At the local level, departments and agencies must strive to develop effective joint working and streamline the adaptation process. At the national level, central government needs to coordinate housing and community care policies to ensure sufficiently funded systems are in place, to define the roles and responsibilities of all partners and to introduce a standardized system for delivering adaptation services.

## Figures and Tables

**Table 1:** Descriptive statistics of partnership for the delivery of adaptations within local authorities.

Which Partners are Working Together for the Delivery of Adaptations in Local Council?					
	Housing Department	The Integration Authority	Social Work	Associated Organizations	Others
*n*	93	15	83	75	26
%	83.0%	13.4%	74.1%	67.0%	23.2%
Are there written guidance specified service entitlements and service processes between the partners?					
	Yes	No			
*n*	87	23			
%	79.1%	20.9%			
How is the effectiveness of current joint work?					
	Very ineffective	Fairly ineffective	Fairly effective	Very effective	
*n*	5	4	57	42	
%	4.6%	3.7%	52.8%	38.9%	

**Table 2:** Crosstabs between self-referral services and delivery outcomes.

Number of Local Authorities Received Different Levels of Self-Referrals					
Number of Adaptations	None (*n* = 43)	1–25% (*n* = 26)	26–50% (*n* = 5)	51–75% (*n* = 9)	Over 75% (*n* = 9)
Fewer than 50	23.3%	15.4%	0.0%	33.3%	0.0%
50–100	41.9%	30.8%	0.0%	44.5%	11.1%
101–150	20.9%	19.2%	0.0%	11.1%	33.3%
151–200	9.3%	3.8%	40.0%	11.1%	33.3%
Over 200	4.6%	30.8%	60.0%	0.0%	22.3%

**Table 3:** Crosstabs between the screening mechanism and delivery outcomes.

	The Use of an Initial Screening Mechanism	
Number of Adaptations	Yes (*n* = 73)	No (*n* = 20)
Fewer than 50	15.1%	30.0%
51–100	28.8%	45.0%
101–150	17.8%	25.0%
151–200	16.4%	0.0%
Over 200	21.9%	0.0%
